# Compact NMR Spectroscopy for Low-Cost Identification and Quantification of PVC Plasticizers

**DOI:** 10.3390/molecules26051221

**Published:** 2021-02-25

**Authors:** Anton Duchowny, Alina Adams

**Affiliations:** Institut für Technische und Makromolekulare Chemie, RWTH Aachen University, Templergraben 55, 52056 Aachen, Germany; Duchowny@itmc.rwth-aachen.de

**Keywords:** plasticizer, PVC, identification, quantification, non-deuterated solvent, low-field NMR spectroscopy

## Abstract

Polyvinyl chloride (PVC), one of the most important polymer materials nowadays, has a large variety of formulations through the addition of various plasticizers to meet the property requirements of the different fields of applications. Routine analytical methods able to identify plasticizers and quantify their amount inside a PVC product with a high analysis throughput would promote an improved understanding of their impact on the macroscopic properties and the possible health and environmental risks associated with plasticizer leaching. In this context, a new approach to identify and quantify plasticizers employed in PVC commodities using low-field NMR spectroscopy and an appropriate non-deuterated solvent is introduced. The proposed method allows a low-cost, fast, and simple identification of the different plasticizers, even in the presence of a strong solvent signal. Plasticizer concentrations below 2 mg mL^−1^ in solution corresponding to 3 wt% in a PVC product can be quantified in just 1 min. The reliability of the proposed method is tested by comparison with results obtained under the same experimental conditions but using deuterated solvents. Additionally, the type and content of plasticizer in plasticized PVC samples were determined following an extraction procedure. Furthermore, possible ways to further decrease the quantification limit are discussed.

## 1. Introduction

The amount of plastics produced worldwide has been increasing steadily in recent decades, with poly(vinyl chloride) (PVC) being the third most produced polymer after polyethylene and polypropylene [1]. PVC products are widely used in many fields of application including consumer products, construction, and packaging materials as well as medical devices. Concomitant with the growth of polymer production also comes a significant increase in the amount of used additives such as antioxidants [2], organic peroxides [3], and plasticizers. In particular, plasticizers account for about one third of the additives [4]. Forecasts predict a rise in the global demand for plasticizers to about 9.75 million tons in 2024 [5]. Plasticizers play an essential role in almost all formulations of polymer products, being especially important for PVC. The plasticizer content in PVC ranges from small amounts up to about 80 wt% for various industrial products [6,7]. Plasticizers are usually larger molecules with molar masses between 200 and 500 g/mol which have bulky or long side groups and serve the purpose to improve the flexibility of the PVC products by lowering the glass transition *T*_g_ of the pure polymer which is about 82 °C.

Due to economic and technical reasons, plasticizers are, in most cases, simply mixed with the polymer material [4]. In contrast to inner plasticizers, these external plasticizers are not chemically bound to the polymer chain and generally tend to migrate out of the product over time or in the presence of solvents. The detected leaching is strongly dependent on the experimental conditions and on the type and amount of plasticizer [8,9,10,11,12,13,14]. As a consequence, developing suitable mathematical models for predicting plasticizer leaching under various experimental conditions is a very challenging task, as also shown by the failure of accelerated tests to predict its long-term migration behavior [15,16]. The modeling is even more complicated for the plasticizer loss in the presence of solvents. This is due to a combined interplay between the migration of the plasticizer in the surrounding liquid and the ingress of the liquid itself into the PVC product at diffusivity rates which strongly depend on the concentration of the plasticizer inside the PVC and on the type of solvent [17].

However, a precise prediction of the behavior of a particular plasticizer under working conditions of a PVC product is of key importance for an improved understanding of the two major issues accompanying the plasticizer loss: 1) the deterioration of the original performance of the PVC products [18,19,20,21] and 2) possible environmental and health risks due to the toxicity of phthalate plasticizers [22,23,24,25]. Due to this, risk assessments and regulations have been introduced concerning the usage of plasticizers in products designed to be in contact with human skin or groceries [26,27,28]. This also led to the development of novel strategies to reduce the plasticizer loss and to design alternative and phthalate-free plasticizers [4,29,30,31].

Only very few studies exist today about the migration of these novel plasticizers and their health risks [14,32,33]. A reliable assessment of the above-mentioned risks requires identifying the type of plasticizer inside a PVC product and quantifying its release under particular experimental conditions. This in turn will help in designing plasticizers with improved properties. In addition, simple, cost-efficient, and reliable ways are needed for controlling, on a regular basis, how far the legal regulations are indeed respected.

Various analytical techniques are nowadays applied in the identification and/or quantification of plasticizers in PVC. They include Fourier-transform infrared (FT-IR) spectroscopy, mass spectrometry (MS), liquid and gas chromatography (LC and GC), thermogravimetric analysis, terahertz spectroscopy, and solid- and liquid-state nuclear magnetic resonance (NMR) spectroscopy [34,35,36,37,38,39,40,41]. Aside from FT-IR, LC, and GC-MS, a recent publication compared the analytical performance of liquid-state NMR spectroscopy conducted at a high magnetic field of 500 MHz and identified NMR as being a primer method able to precisely discriminate all investigated plasticizers [37]. This result is further supported by the identification of seven plasticizers in medical devices followed by quantification of their concentrations by adhering to a defined measuring procedure of the high-field NMR method in deuterated solvents [42]. Despite being, nowadays, an indispensable method for structural determination in chemistry, the applicability of high-field liquid-state NMR for the identification and eventual quantification of plasticizers in PVC was, up to now, restricted to a few dedicated studies largely from academia [38,42]. This is because the high-field NMR devices are expensive, need to be operated by skilled personnel in special facilities, and require the usage of deuterated solvents, which are more costly than the corresponding non-deuterated solvents. Hence, high-field NMR is usually not the method of choice for low-cost routine analysis in industry, in a medical environment, and even in academia.

However, the development of compact NMR instruments with open and closed geometries has opened new perspectives in many fields of activities [43,44,45,46,47,48,49,50,51]. Such NMR devices working at a low magnetic field, in the range of 40 to 60 MHz, are commercially available at low prices and can be operated by non-experts. Having a small size and being light, they can be placed in a synthesis laboratory on a bench or under a fume hood, near a production line, or in a corner in a hospital. Furthermore, a large variety of experimental NMR methods, including one-dimensional ^1^H and ^13^C spectroscopy, 2D spectroscopy, and relaxation, are readily implemented to work at low-field NMR. As a consequence, compact low-field NMR has become an excellent alternative to high-field NMR for a large variety of investigations. In particular, low-field NMR spectroscopy is well suited for the detailed structural characterization of small molecules and reaction monitoring [44,46]. The application of low-field NMR spectroscopy for the study of larger and more complex molecules has started more recently. This is because the analysis of the corresponding spectra is far more challenging than for smaller molecules due to a stronger overlap of the resonances than in the high field. Comparisons with the corresponding liquid spectra from high-field NMR and/or a combination with chemometrics or other analytical methods are useful and often the needed method for a reliable signal identification and assignment [45,52,53,54,55].

**Figure 1:**
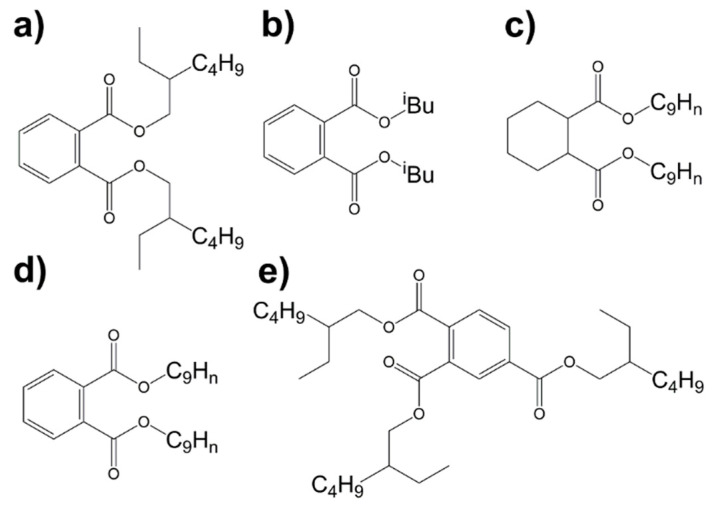
Structure of the investigated plasticizers in the current study: (**a**) diethylhexyl phthalate (DEHP), (**b**) diisobutyl phthalate (DIBP), (**c**) diisononyl cyclohexane-1,2-dicarboxylate (DINCH), (**d**) diisononyl phthalate (DINP), (**e**) tris(2-ethylhexyl) trimellitate (TOTM).

In this work, the applicability of low-field NMR spectroscopy for the identification and quantification of PVC plasticizers is, for the first time, investigated and demonstrated with the help of five different plasticizers (Figure 1). In addition, a novel experimental protocol is proposed for gaining the needed information in a fast way and without the need for deuterated solvents. Moreover, possible methodological and hardware improvements to further lower the quantification limit are discussed. These traits make the introduced approach particularly interesting as an alternative to high-field NMR and for routine quality control in various environments. Furthermore, it could be used for the identification and quantification of additives for other polymer materials.

## 2. Results and Discussions

### 2.1. ^1^H NMR Spectroscopy

^1^H NMR spectroscopy is an appropriate analytical method to analyze PVC plasticizers as it can differentiate between the signals given by various functional groups and their intensities are directly related to the amount of plasticizer inside the NMR tube. According to results acquired at high-magnetic fields in deuterated solvents [42,56], the ^1^H NMR spectra of various plasticizers contain peaks in the aromatic region around 7 ppm, between 3 and 4 ppm for the α-CH_2_ groups next to the ester bond, and at around 1 ppm for aliphatic CH_2_, while the CH_3_ chain ends appear at around 0.8 ppm. An exception to this is DINCH which has a cyclohexene dicarboxylic acid core instead of phthalic acid and consequently shows no peak in the aromatic region of the spectrum. These features show that various plasticizers can be well discriminated by using characteristic proton resonances.

One can thus anticipate that a discrimination of plasticizers would also be possible using low-field NMR spectroscopy even if the resonances would be less separated. Furthermore, given that the characteristic proton resonances are located in a spectral range above 2.5 ppm, one could argue that non-deuterated solvents, which give signals outside the range of interest, can be used to acquire the proton spectra instead of deuterated solvents. One example of such solvent is n-hexane which is reported to be a good solvent for plasticizers [57] and gives proton signals under 1.3 ppm, at positions largely independent of the presence of other solvents [58]. A methodology using non-deuterated solvents is especially attractive in the view of the strongly reduced costs as compared to the case when deuterated solvents are employed for routine analyses. It is also more appropriate for investigating plasticizer leaching under real conditions.

**Figure 2:**
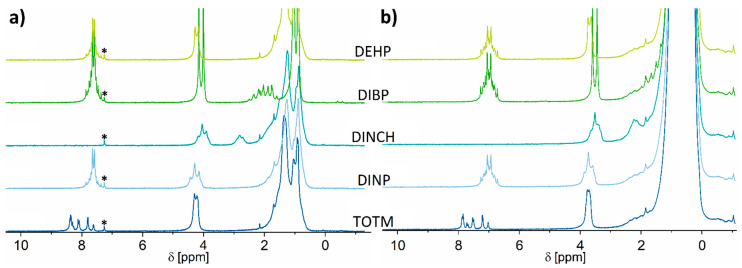
40 MHz ^1^H NMR spectra of the investigated plasticizers at concentrations of 10 vol.% in (**a**) deuterated chloroform and (**b**) non-deuterated n-hexane. Spectra have been referenced to the residual deuterated chloroform (signal marked with asterisk at 7.26 ppm) and n-hexane (0.8 ppm) peaks, respectively. The difference in the chemical shift of ~0.5 ppm in the signals in the two subfigures can be attributed to solvent effects. Most of the spectra are zoomed in on for a better view of the signals above 2 ppm.

Typical proton low-field NMR spectra of various plasticizers dissolved in deuterated chloroform as well as in non-deuterated hexane are depicted in Figure 2a,b and in full scale in Figure S1. One can clearly observe from Figure 2a that the spectral region below 3 ppm is very crowded, and the signals are largely similar among the various plasticizers, except for DIBP. Nevertheless, this is not an impediment as the signals above 3 ppm are well separated and can be used for the purpose of identification and quantification. This observation is in agreement with the results previously reported [42,56]. Exactly these spectral features are also advantageous when a plasticizer is dissolved in a non-deuterated solvent. This is demonstrated in Figure 2b by the typical low-field ^1^H NMR spectra of the investigated plasticizers in non-deuterated n-hexane for a plasticizer concentration of 10 vol.% (94.4–103.9 mg mL^−1^) and in Figure S2 for varying concentrations down to 0.1 vol.% (0.97 mg mL^−1^). As expected, the solvent shows strong signals at lower ppm values. Obviously, the resonances below 2 ppm in Figure 2b are covered by the strong n-hexane peak and, thus, cannot be used for quantification. However, the spectra acquired in deuterated chloroform and non-deuterated n-hexane are highly similar, being above 3 ppm, except the residual chloroform peak and differences in chemical shift due to solvent effects. Thus, the aromatic and α-CH_2_ regions can be used to identify and quantify PVC plasticizers. Even at a plasticizer concentration as low as 1 vol.% and below, characteristic resonances of the various plasticizers can be identified in this spectral range in the presence of the non-deuterated solvent which poses no impediments by its strong signal in the low-ppm region (Figures S2 and S3). Moreover, the ^1^H low-field NMR spectra depicted in Figure 2 conveniently allow the identification of the various plasticizers with the help of the specific spectral features, despite the used magnetic field being a factor 10 lower than in [42].

The aromatic spectral region around 7 ppm indicates that one can easily differentiate DINCH from all the other plasticizer types by the lack of signals in this region. Furthermore, TOTM shows a completely different spectral pattern at this position compared to all the other investigated plasticizers. This specific signal can be used for its identification. The observed spectral pattern is because TOTM is a derivate of trimellitic acid instead of phthalic acid, which has three rather than two carboxylic acid side groups. This leads to the strong splitting of the aromatic peak. Additionally, as TOTM possesses one more substituent at the aromatic ring, less hydrogen atoms contribute to the aromatic signal. DINP, DIBP, and DEHP show similar peak shapes in this spectral region, which makes their identification impossible when using only this signal. However, due to the structural differences in their aliphatic side chains, the peaks between 3 and 4 ppm have different shapes and intensities compared to the signals around 7 ppm. The specific characteristics of the three plasticizers in this region can also be used for their identification. In particular, DIBP shows the most distinctive and intense signal. Its peak at 3.5 ppm has a doublet with a J-coupling constant of 6.5 Hz due the single proton bound to the tertiary carbon atom at the β-position of the isobutylic group. DEHP also shows a doublet, which is shifted towards the lower field by 0.19 ppm and shows a significantly lower J-coupling constant of 4.5 Hz when compared to the DIBP doublet.

DINCH and DINP are mixtures of plasticizers with several iso-nonyl chains, causing the signals in this spectral region to be relatively broad. Despite having a similar shape, the signal of DINCH is shifted to the higher field by 0.23 ppm, allowing further differentiating them.

From the above findings, it becomes clear that the analysis of the peak positions, their shapes, and their intensities in the aromatic and ester regions of the ^1^H spectrum delivers enough information to identify each of these five plasticizers. This type of identification, however, could be more complicated, especially at a low magnetic field, when investigating mixtures of various plasticizers due to the overlap of the resonances of interest. In this case, the use of ^13^C spectra may be needed (see Section 2.2).

Following the identification of the plasticizer type, quantifying its concentration is the next step. Proton NMR spectroscopy is generally well suited for this purpose as the integral of a signal is directly proportional to the number of protons contributing to it and, through that, is directly proportional to the concentration of the investigated sample. The procedure applied in [42] uses an internal reference compound in a coaxial tube inserted into the 5 mm NMR tube containing the plasticizer solution to be measured. The concentration of the used reference compound was a priori calibrated with a plasticizer solution of known concentration. This procedure is common in liquid-state NMR and can be also implemented at low fields but leads to a reduced signal intensity due to the decreased volume of the sample of interest and possibly to peak distortions as well.

**Figure 3:**
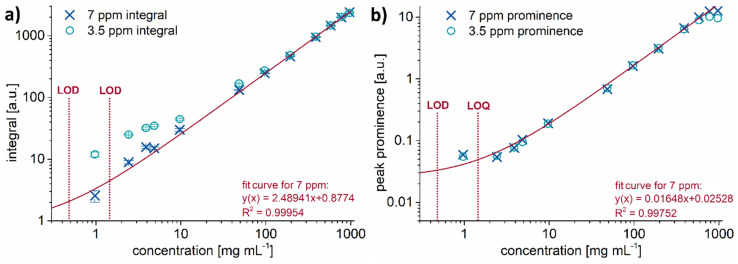
(**a**) Integral and (**b**) peak prominence of the ester (3.5 ppm) and aromatic (7 ppm) peak regions of DINP solutions using non-deuterated n-hexane as a solvent. Concentrations range from 0.97 to 970 mg mL^−1^. The continuous lines are the fit results for the signal at 7 ppm showing the excellent linear correlation between the NMR integral (**a**) or the peak prominence (**b**) and the plasticizer concentration as demonstrated by the R^2^ value. The apparent curvature of the linear fit is due to the log–log scaling of the axes.

To overcome these issues, we generated an external calibration curve by correlating the integral value of the signal of interest to the plasticizer’s concentration. This procedure was applied for all investigated plasticizers in the whole range of studied concentrations. Figure 3a shows, exemplarily for DINP, the dependence between the known plasticizer concentration and the corresponding integral of the two signals, which could, in principle, be used for chemical identification. The integral of the aromatic peak (around 7 ppm) shows a linear behavior with the plasticizer concentration. The ester peak at around 3.5 ppm, however, shows a slight offset from the ideal linear trend which becomes more pronounced on a logarithmic scale with decreasing concentrations. This can be explained by an additional signal given by the solvent’s ^13^C satellite peaks which, at a lower plasticizer concentration, start to overlap with this spectral region. ^1^H-^13^C decoupling techniques come standard with modern compact NMR spectrometer but were not available for the 40 MHz instrument used for this study. If a quantification with the aromatic region is not applicable—like in the case of DINCH—the utilization of a different suitable solvent such as benzene or chloroform would move the strong solvent peaks away from the region of 3.5 ppm, hence abolishing overlapping of solvent and ester peaks. Alternatively, spectral deconvolution techniques could be applied at this point to numerically decrease the error introduced by overlap. Although powerful, spectral deconvolution requires the operator to have the know-how and experience in that field. To keep the proposed quantification methodology simple, we determined the peak prominence in addition to the integral to serve as a compensation for overlapping peaks.

Figure 3b shows that the peak prominences follow a linear trend with the exception of the data point of the lowest concentration. This is below the determined limit of quantification for DINP measured with four scans and thus is strongly influenced by noise. Additionally, the data points for the peak prominence of the 80 and 100 vol.% solutions also do not follow the linear trend. This can be explained by the high, sirup-like viscosity of the pure plasticizer, which causes the Free Induction Decay (FID) to decay more rapidly, resulting in broader, instead of taller, peaks. This behavior is also reflected in the samples’ spin–spin relaxation times as exemplarily shown for TOTM and DIBP in Figure S4. Thus, for quantification purposes, the aromatic peak region around 7 ppm is preferable. If not applicable, like in the case of DINCH, the peak prominence around 3.5 ppm can be utilized as an alternative as it also shows a strongly linear correlation with the plasticizer concentration.

The determined limit of detection (LOD) and limit of quantification (LOQ) values of every plasticizer are highly influenced by the peak structure in the NMR spectrum. Hence, they vary for every individual plasticizer and the used ppm range for this purpose. Consequently, both limits are higher for TOTM in Table 1, caused by the molecular features discussed above. These features effectively lower the signal-to-noise ratio for this plasticizer in the aromatic spectral range, which was used for the determination of the LOD and LOQ. However, when analyzing the ester region of the spectrum, the signal intensity of TOTM would be higher compared to the other plasticizers as there are three, rather than two, ester groups in this molecule.

### 2.2. ^13^C NMR Spectroscopy

Nowadays, liquid-state ^13^C NMR spectroscopy at a high magnetic field is often the method of choice in the structural characterization of complex and larger molecules owing to its lack of homonuclear coupling and broader signal dispersion compared to ^1^H NMR. Liquid-state ^13^C spectra of various plasticizers recorded at a high magnetic field are reported in various sources but, to our knowledge, never in a systematic way towards comparison and quantification and never at a low magnetic field. Therefore, we investigated, for the first time, the applicability of low-field ^13^C NMR liquid-state spectroscopy for the identification of plasticizers and quantification of their concentration.

**Figure 4:**
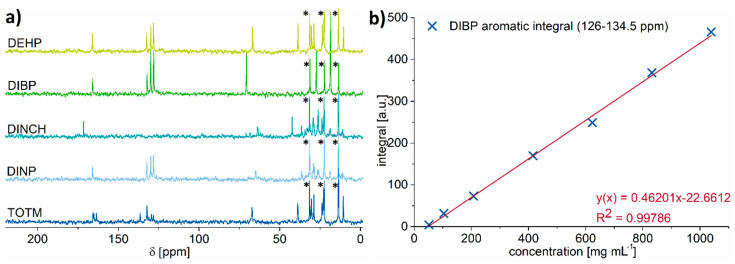
(**a**) ^13^C low-field NMR spectra recorded with a plasticizer concentration of 60 vol.% in non-deuterated n-hexane (signals marked with asterisks). The asterisks correspond to the signals from n-hexane. (**b**) Integral versus concentration plot showing excellent linear behavior as illustrated by the aromatic region of DIBP.

Due to the low natural abundance of ^13^C, the acquisition of the spectra for obtaining a reasonable signal-to-noise ratio lasts much longer than the corresponding ^1^H spectra. For investigating how far ^13^C spectroscopy at a low field strength is applicable to everyday practice, the acquisition time of recording the spectra was set to around 32 min. This was achieved by accumulating 128 scans with a repetition delay of 15 s. Figure 4a exemplarily depicts the ^13^C low-field NMR spectra of all studied plasticizers in n-hexane at a concentration of 60 vol.%. In addition, Figure S5 shows the acquired ^13^C spectra of DIBP, ranging from 10 to 100 vol.%.

While n-hexane shows three distinct signals at around 14, 23, and 32 ppm (marked with asterisks in Figure 4a and Figure S5), all the other signals belong to the plasticizers. They show a large dispersion over almost 180 ppm and all are well observable, even under the used experimental conditions. Furthermore, the signals of all plasticizers are outside the range where dissolved PVC would have its own signals (from about 44 to 49 ppm and from about 55 to about 58 ppm) [38]. This means that one could perform the measurements directly on the dissolved plasticized PVC sample without the need for removing the polymer or performing extraction studies.

Analyzing the spectral range of the carboxylic carbon atom between 160 and 175 ppm already allows identifying DINCH and TOTM. This is because the ring structure of these plasticizers differs from the phthalate-based ones. As a result, DINCH shows a peak at 171.54 ppm and TOTM shows three peaks at 163.49, 165.02, and 165.61 ppm, whereas the other plasticizers exhibit one peak at roughly 166 ppm. As these differences in the carboxylic peak region arise from differences in the aromatic core of these plasticizers, the same conclusion can be drawn when analyzing the aromatic part of the spectrum between 127 and 137 ppm. DINCH shows no signals in this region, whereas TOTM exhibits a more complex peak structure compared to the signals of the other three molecules.

DEHP, DIBP, and DINP all possess different aliphatic side chains. Therefore, these components can be identified by analyzing the signal of the carbon atom at the α-position of the side group between 60 and 71 ppm. Here, DIBP shows the highest chemical shift with 70.66 ppm. DEHP, on the other hand, has its peak at 66.87 ppm, followed by DINP which has the smallest chemical shift in this spectral region and shows two peaks at 64.72 and 65.05 ppm. In theory, DINCH and DINP should have an identical peak pattern here. However, as both chemicals are mixtures with different iso-nonyl side chains, this is not true. In this case, DINP only shows one signal for the α-position of the side chain and no signal around 38–43 ppm where an aliphatic tertiary carbon atom would appear. This fact suggests that the examined DINP contains more n-nonyl side chains, whereas for DINCH, the aliphatic groups are a mixture of several iso-nonyl groups.

As a conclusion, the investigated plasticizers can easily be distinguished from others even at a low magnetic field strength with a simple comparison of the ^13^C spectra. However, this will not be feasible during the half an hour measuring time when very low amounts are present. Furthermore, their quantification using ^13^C NMR is challenging. The relaxation times *T*_1_ of ^13^C nuclei usually have higher values compared to ^1^H. This circumstance and the low ^13^C natural abundance of 1.1% translate to longer measuring times. Moreover, ^13^C liquid-state spectra are, in most cases, recorded using shorter recycle delays than those dictated by the ^13^C longitudinal relaxation times *T*_1_. Thus, the recorded spectrum is not quantitative and the signal integral will be also affected by the nuclear Overhauser effect (NOE) enhancement. This means that the peaks’ integral does not necessarily correspond to the amount of contributing ^13^C nuclei from the molecule. The signal intensity of the observed carbon nucleus will be boosted depending on the amount of hydrogen nuclei close to it as these can transfer their nuclear polarization to the carbon. Therefore, the spectra shown in Figure 4 are not quantitative in the way ^1^H spectra are, meaning that, e.g., if one peak in the spectrum is double in integral compared to a second, it is not necessarily the case that this signal is produced by twice as many nuclei.

However, following the same procedure as applied for proton spectra and using the same series of samples, the correlation between the integrals of particular ^13^C signals and the known plasticizer concentrations can be investigated. A linear behavior between the plasticizer’s concentration and the peak integral is obtained for various signals as exemplarily depicted for DIBP (Figure 4b). These curves can then be employed for any further quantification of the plasticizer concentration. The use of only 128 scans enables identification of plasticizers at concentrations as low as 50 mg mL^−1^, but they are, however, not enough for a reliable quantification of concentrations under 100 mg mL^−1^ due to the high noise level. Both values could be further improved at the cost of longer measurement times.

### 2.3. Test of the Proposed Method

**Figure 5:**
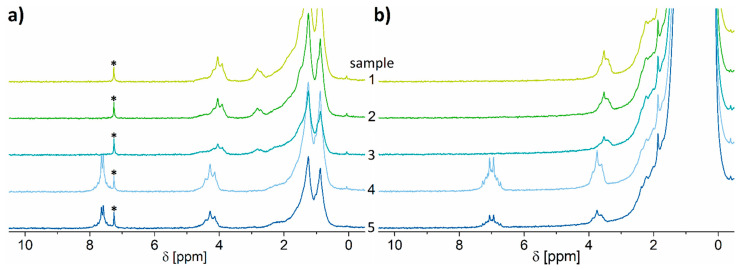
^1^H spectra of the unknown PVC samples 1–5 after the extraction in (**a**) CDCl_3_ (residual non-deuterated solvent marked with asterisks) and (**b**) non-deuterated n-hexane.

To test the reliability of the proposed method for the identification and quantification of plasticizers using ^1^H low-field spectroscopy in the presence of non-deuterated solvents and with the help of external calibration, plasticizer extraction experiments with five plasticized PVC samples with unknown histories using both deuterated and non-deuterated solvents were performed. Each extraction, as described and validated in the literature [57], was repeated three times using deuterated chloroform and non-deuterated n-hexane. Figure 5 shows the obtained ^1^H spectra.

Identification of the plasticizer in the samples is convenient, even at a low magnetic field strength, as the spectra of the pure components were available from the calibration step. Given the specific spectral features of each plasticizer, the results from Figure 5 indicate that all samples contain only one type of plasticizer. In particular, the samples 1 to 3 can be identified as samples containing DINCH, while the samples 4 and 5 can be identified as samples containing DINP. Integrating the spectrum in the same ppm region as employed for the calibration curve yields the plasticizer concentration in the sample solution in the tube. The obtained results are shown in Table 2.

Since DINCH does not contain aromatic structures, its signal around 3.5 ppm was used for analysis. This ppm range shows some overlap with the hexane peak as the concentration of the plasticizer in the extraction liquid is relatively low. In order to increase the accuracy of the DINCH quantification, we determined the peak prominence and full-width-half-maximum (FWHM) to compensate for the hexane overlap.

The results in Table 2 would indicate, at a first glance, a large standard deviation of the analytical measurement. Re-measuring the same samples yielded a highly similar spectrum and plasticizer content. The relative error of the integral between several measurements of the same sample tube with plasticizer concentrations between 5 and 30 mg mL^−1^ is about 0.5–0.7%, which is much lower than the errors in Table 2. The reason for the detected differences is due to a heterogeneous distribution of the plasticizer in the PVC sheets. The fact that samples taken in the middle of the sheet contained less plasticizer compared to the edges indicates that the plasticizer has already migrated to the outside of the polymer structure. A comparison of ^1^H-NMR spectra measured at the low field and at 400 MHz on the same sample tubes validated the fact that samples taken from the same PVC sheet indeed contained different amounts of plasticizer. This further indicates that the precision of the measurement is high, but the local plasticizer content in different areas of the PVC sheet varies.

Ascertaining the plasticizer content gravimetrically to cross-check the NMR results yielded a plasticizer content between 7.2 and 11 wt.% lower compared to the extraction method. This result suggests that either the plasticizer was not fully extracted, or the solvent could not completely be removed from the PVC sample after several days of vacuum drying. A second extraction step with just enough solvent to cover the sample, however, showed no plasticizer signal at all, even with a larger number of scans. Kastner et al. [14] reported complex interactions between the solvent and plasticized polymer, indicating that gravimetric analysis, though simple in preparation and execution, is not suitable for plasticizer quantification without further methodical modifications. Results of the gravimetric analysis are shown in Table S1 and S2.

### 2.4. How to Further Improve the Low-Field NMR Identification and Quantification

The most accessible method of identifying and quantifying plasticizers in PVC products, which offers reasonable precision with a lower consumption of time, costs, and workforce, was aimed for. Possibilities to improve this method are plentiful but they will add an additional step in the sample preparation, costs, or the time needed for an analysis. The easiest improvement would be the implementation of ^13^C-decoupling in the ^1^H spectra. This option comes standard in today’s benchtop NMR spectrometers with a carbon channel but was not available with the 40 MHz instrument used in this study as it is one of the first-generation devices. ^13^C-decoupling would have a noticeable effect especially on the n-hexane peak and would vastly enhance the spectral resolution at low plasticizer concentrations. Figures S2 and S3 exemplarily visualize this effect on various plasticizers. The ^13^C satellite peaks of hexane appear between 2 and 2.5 ppm as well as below 0 ppm. At low plasticizer concentrations, the satellite peaks have a similar intensity to the analyte and increase the width of the already pronounced n-hexane peak.

Another option to improve the analysis outcome in terms of identification followed by quantification would be to increase the signal-to-noise ratio (SNR). For the extraction method, we used plenty of solvent compared to the mass of PVC. Hence, the plasticizer concentration of the extract was low (1–3 wt.%). The easiest way to improve the SNR would be to decrease the amount of solvent used for extraction. However, in this case, one runs the risk of an incomplete extraction. Alternatively, one could let the solvent evaporate after the extraction time and re-solve the plasticizer with just enough solvent to fill the sensitive region of the NMR spectrometer. In this way, the concentration and therefore the signal strength of the examined sample can drastically be increased which facilitates the plasticizer identification. Having a relative vapor pressure of a maximum of 60 Pa (DINP) compared to 162 hPa for n-hexane (209 hPa for chloroform) at 20 °C makes it unlikely to lose a significant amount of plasticizer during solvent evaporation. A second alternative to increase the SNR for given experimental conditions in terms of the magnetic field strength and temperature is by increasing the number of scans. This in turn leads to an increase in the experimental time. Nevertheless, in this way, the LOD and LOQ can be further decreased.

**Figure 6:**
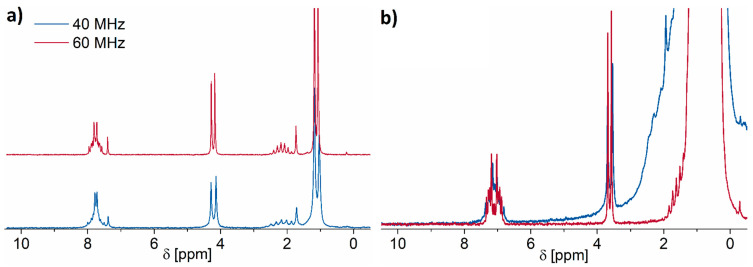
16.67 mg mL^−1^ DIBP solution in (**a**) CDCl_3_ and (**b**) n-hexane measured at 40 and 60 MHz. The left graphic shows a stacked plot as the differences between the spectra are hardly visible when displayed superimposed.

Furthermore, benefits can be gained by eliminating the peak overlap. This can be achieved by using deuterated solvents such as d-chloroform, which we used in the solvent extraction method as an alternative to n-hexane. Most of these, however, are more costly and thus the choice of solvents is limited. Technical n-hexane is around 10–15 times less expensive than d-chloroform, which can be considered as one of the low-priced deuterated solvents. As the results of this work have shown, there is no drawback in terms of identification and quantification of plasticizer solutions if n-hexane is being utilized. However, deuterated solvents can be beneficial if, e.g., mixtures of multiple plasticizers are present, making their identification more challenging. As shown in Figure 6a, all three spectral regions of interest (aliphatic, ester, and aromatic) are analyzable and separated by the baseline when d-chloroform is being used as a solvent.

Alternatively, both the signal-to-noise ratio and peak overlap can be improved by increasing the magnetic field strength of the NMR device. In order to keep all the benefits benchtop NMR offers for routine analysis, we compared spectra acquired with the Spinsolve 40 Carbon, which was used in this study, with those acquired using a Spinsolve 60 ULTRA working at a magnetic field of 60 MHz, rather than 40 MHz. The effect of increasing the magnetic field strength is shown in Figure 6b where a sample containing a DIBP concentration of 16.67 mg mL^−1^ is measured on both devices with four scans and a 15 s repetition delay. The result indicates that the 60 MHz spectrometer delivers baseline separated peaks with the n-hexane solution, which is a great benefit compared to the 40 MHz spectrum. When extracting the plasticizer with deuterated chloroform, however, upgrading to the 60 MHz device hardly shows to be beneficial.

According to the regulation (EC) No 1907/2006 of the European Parliament and of the European Council [59], a relevant plasticizer present in a polymer by more than 0.1% per weight must undergo a chemical safety assessment. Such a plasticizer content in a PVC product leads to a concentration of 0.05 mg of plasticizer solved in 1 mL of n-hexane after the solvent extraction has been performed, as described in this work. As depicted in Table 1, this concentration is lower than the LOQ achieved with the 1 min measuring time by ^1^H NMR spectroscopy. In order to determine concentrations at this legal threshold with the low-field NMR method, the number of scans should theoretically be increased to around 400 in order to increase the SNR. Measuring a 0.05 mg mL^−1^ solution of DIBP with 256, 512, 1024, and 2048 scans with a 7 s repetition time led to the result that the LOQ could only be reached with 1024 scans in combination with an advanced baseline correction. Nevertheless, Figure S6 shows that distinct qualitative spectral features are still noticeable without implementing more sophisticated methods. Thus, slightly adjusting the straightforward low-field NMR method proposed in this work with further improvements discussed in this section can meet the criteria given by the European Union, even for less experienced users.

### 2.5. Low-Field NMR versus Conventional High-Field NMR

Given that low-field NMR hardware is, by far, more affordable compared to high-field NMR hardware and the costs for maintenance, extra personnel, and facilities are negligible, the results shown in the previous sections indicate that the proposed low-field NMR analysis is a low-cost alternative for the study of PVC plasticizers. The analysis costs at a low field can be further decreased with the use of non-deuterated solvents. However, limits of identification and quantification for plasticizers are higher when comparing our approach with the reported results in [42], which employed high-field NMR.

With the data provided in [42], a minimum plasticizer content in PVC was calculated, which can still be detected by high-field NMR after solvent extraction within 20 min of the measuring time, and we compared this value to the reported low-field NMR results. The minimum plasticizer content in PVC detected at the low field within 1 min, a measuring time which is 20 times lower than in the high field, is only higher by a factor 3 compared to the high-field value (0.336 and 0.96 wt%). This factor 3 relates primarily to the combination of two effects: the size of the sensitive volume and the amount of solvent used for the extraction step.

Advantageous for the low-field method is the larger sensitive volume (0.4 mL) compared to the high-field NMR magnet which results in higher absolute plasticizer contents at identical concentrations. More precisely, the high-field NMR magnet has a sensitive volume of 0.2 mL, which is further decreased to 0.167 mL due to the employed coaxial insert [42]. Furthermore, this work successfully lowered the detectable plasticizer content by reducing the amount of solvent in the extraction step to the lowest ratio reported in the literature. This leads to the phenomenon that the LOD of a plasticizer solution at the high field is lower by a factor of roughly 1000 (42 µg mL^−1^ at the high field versus 48 mg mL^−1^ at the low field for DINP), but the minimum detectable plasticizer content in PVC is only lower by a factor of 3.

## 3. Materials and Methods

### 3.1. Samples

All solvents and plasticizers investigated in this study were purchased from Sigma-Aldrich and used without further purification. Dilution series with plasticizer concentrations ranging from 0.1 to 100 vol.% (0.97–1039 mg mL^−1^) were prepared with deuterated chloroform and non-deuterated hexane using Eppendorf pipettes. A total volume of the plasticizer/solvent mixture of 0.5 mL was then filled into a standard 5 mm NMR tube. ^1^H-NMR spectroscopy measurements revealed that no detectable amount of additive from the pipette’s plastic tip was extracted during the time of filling the utilized solvents into the NMR tubes. The NMR tube was then tightly sealed to prevent solvent evaporation during measurements and storage. In addition, the tube’s filling level was marked after filling to serve as a control feature.

Plasticized PVC samples with unknown histories were used to test the proposed procedure and for identifying the plasticizer type and quantifying its amount with the help of solvent extraction procedures. For this, each available PVC sample was cut in small pieces and carefully weighted. Extraction was conducted at room temperature by using 130–300 mg PVC and 2.6–6 mL (roughly 20 times the amount of the PVC sample) of deuterated chloroform or non-deuterated hexane for 24 h according to [57]. Proton NMR spectra were recorded for the extracted solutions to obtain the plasticizer concentration *c*_sample_. The total mass of extracted plasticizer, as defined by equation (2), can be calculated by transposing equation (1). This value is then required to determine the mass percentage of plasticizer in the PVC sheet. Following the extraction step, the sample sheets were dried under vacuum for 24 h and weighted again to additionally analyze the amount of plasticizer loss gravimetrically.

(1) $$c_{\mathrm{sample}}=\frac{m_{\mathrm{plasticizer}}}{m_{\mathrm{solvent}}+m_{\mathrm{plasticizer}}}$$

(2) $$m_{\mathrm{plasticizer}}=\frac{m_{\mathrm{solvent}}*c_{\mathrm{sample}}}{1-c_{\mathrm{sample}}}$$

### 3.2. NMR Experiments

**Figure 7:**
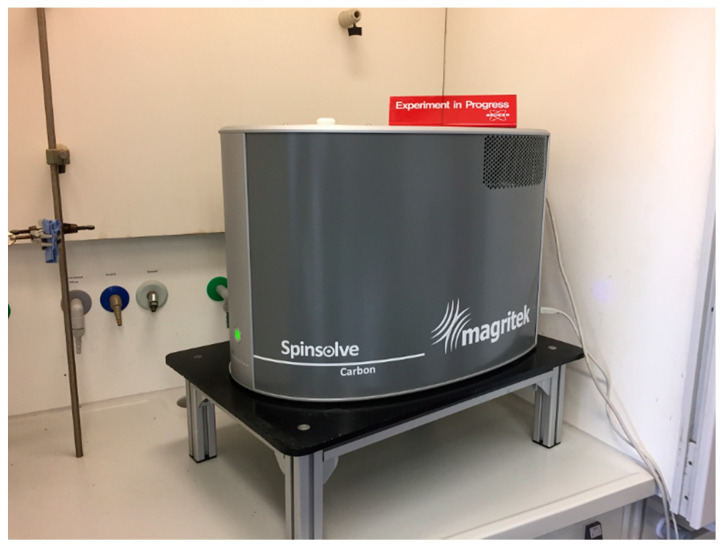
Photo of the utilized Spinsolve placed inside a fume hood in a chemistry laboratory.

The NMR experiments were performed on a Magritek Spinsolve 40 Carbon (Figure 7) working at a frequency of 43 MHz for protons and 11 MHz for ^13^C and at a constant magnet temperature of 28 °C. For selected samples, ^1^H spectra were measured also using a Magritek Spinsolve 60 ULTRA working at a frequency of 60 MHz for protons and at a constant temperature of 26.5 °C. Before each measurement, the magnetic field was shimmed to a linewidth of less than 0.5 Hz at half peak height using a 90/10 D_2_O/H_2_O sample for 40 MHz and a linewidth of less than 0.3 Hz at half peak height was achieved at 60 MHz using a 95/5 D_2_O/H_2_O sample as specified by the manufacturer. ^1^H and ^13^C spectra as well as ^1^H spin–lattice relaxation times *T*_1_ were measured for dilution series for every plasticizer. All ^1^H spectra were acquired using 4 scans and the device’s standard repetition time of 15 s, which is longer than the corresponding 5× *T*_1,_ with *T*_1_ being between 1 and 2 s at most for all plasticizers. Thus, the total measuring time for a spectrum was 1 min. This number of scans was chosen to keep the experimental time short. This is especially of interest if a daily analysis routine with large sample quantities is planned.

All spectra were referenced to the distinct solvent peak present in the mixtures. *T*_1_ were acquired with the inversion recovery method, accumulating 4 scans at inversion times ranging from 1 ms to 5 s. Knowing the samples’ relaxation times permits the manual reduction in the repetition time without suffering a signal intensity loss. However, for the purpose of simplicity and the easy application of the method for non-NMR users, we decided to employ the standard repetition time of 15 s implemented in the Spinsolve software. All ^13^C spectra were measured using proton decoupling during acquisition by accumulating 128 scans also with a repetition time of 15 s.

Due to reproducibility concerns, all NMR measurements conducted for the calibration were performed 5 times for all plasticizers. Plasticizer signals in another spectrum region different to that of the solvent were integrated for quantifying the plasticizer content in a particular sample.

For determining the limit of detection (LOD) and the limit of quantification (LOQ) [60] of the low-field NMR method, phase and baseline corrections as well as a line broadening of 0.3 Hz were applied to the spectra. The peak integral of the aromatic peak region was plotted against the solution’s concentration and then a linear fit curve was used to determine the LOD and LOQ. Knowing the instrument’s sensitive volume allows calculating the corresponding weight percentages of a plasticizer in a PVC material in order to detect or quantify it by the solvent extraction method.

## 4. Conclusions

This work evaluates, for the first time, the applicability of ^1^H and ^13^C low-field NMR spectroscopy for the identification of various PVC plasticizers in solution and the quantification of their amount. While the standard way of conducting such studies is by dissolving the plasticizers in expensive deuterated solvents, our work demonstrates that the same information can be obtained with the help of suited non-deuterated solvents, given that they have their signals outside the range of interest. Furthermore, the same non-deuterated solvents can also be used for the extraction step of plasticizers from PVC products.

The identification of plasticizers was conducted with the help of specific ^1^H and ^13^C resonances, which are well separated even at the low field. These signals were then used for quantification purposes in conjunction with a priori established correlation curves. The correlation curves were established between the integral of the signals of interests in both the ^1^H and ^13^C and the known concentration of plasticizer in solution. ^1^H NMR spectroscopy showed to be the most promising tool in terms of the minimum plasticizer content to be identified and quantified in a short period of time due to the much higher SNR than ^13^C NMR. More precisely, low-field ^1^H spectroscopy enables the identification and the quantification of plasticizer concentrations as low as 2 mg mL^−1^ in solution, corresponding to ~3 wt% in a PVC product, within one minute of the measurement time. The suitability of the proposed method using non-deuterated solvents is demonstrated by comparisons with the spectra recorded on the same plasticizer dissolved in a suited deuterated solvent and by identifying the type of plasticizer extracted from the PVC of samples with unknown histories and quantifying its amount.

Analyzing the minimum plasticizer content in PVC products requested by the European Union is achievable, if the straightforward approach discussed in this work is refined with the methods discussed in Section 2.4. Thus, the combination of low-field NMR with non-deuterated solvents offers a very cost-effective, yet powerful method complementary to high-field NMR, LC, FTIR, or GC-MS, making it well suited for routine quality analysis of large numbers of plasticized PVC samples.

## Figures and Tables

**Table 1:** Detection and quantification limits of examined plasticizers dissolved in non-deuterated n-hexane and measured at 40 MHz within 1 min. Both limits are given in concentrations for the analyzed solution and in amounts a PVC material would need to contain to achieve these concentrations after a solvent extraction. The aromatic peaks’ integral was used to determine detection and quantification limits for all plasticizers except DINCH, where the ester peaks’ prominence at 3.5 ppm was selected.

	DINP	DIBP	DEHP	TOTM	DINCH
LOD [mg mL^−1^]	0.48	0.42	0.57	1.52	0.63
LOQ [mg mL^−1^]	1.45	1.25	1.70	4.58	1.90
LOD [wt% in PVC]	0.96	0.83	1.13	3.05	1.27
LOQ [wt% in PVC]	2.89	2.49	3.39	9.15	3.80

**Table 2:** Results of the extraction experiment. The determined concentration of the extraction solution has been converted to wt.% of plasticizer the PVC initially had, assuming all of the plasticizer had been extracted. Each extraction step was performed 3 times for each investigated PVC sheet by using samples from different positions in the sheet.

Sample	Identified Plasticizer Type	Determined Plasticizer Content [wt.%] from CDCl3 Extraction		Determined Plasticizer Content [wt.%] from n-hexane Extraction	
1	DINCH	38.49	±1.93	42.69	±1.64
2	DINCH	31.68	±1.83	34.14	±1.19
3	DINCH	15.92	±3.63	17.97	±2.60
4	DINP	40.94	±0.10	43.26	±3.98
5	DINP	23.85	±0.95	22.64	±1.38

## Data Availability

The data presented in this study are available on request from the corresponding author.

## Supplementary Materials

The following are available online, Figure S1: 40 MHz ^1^H NMR spectra of the investigated plasticizers at concentrations of 10 vol.% in (a) deuterated chloroform and (b) non-deuterated n-hexane without zoom. Spectra have been referenced to the residual deuterated chloroform (signal marked with asterisk at 7.26 ppm) and n-hexane (0.8 ppm) peaks, respectively. Figure S2: 40 MHz ^1^H NMR spectra of DINP in non-deuterated hexane at varying concentrations. All spectra have been referenced to the signal of the n-hexane (0.8 ppm) peak. The specific resonances at around 7 ppm can be observed even at concentrations as low as 0.1 vol.% with only 4 scans. Figure S3: 40 MHz ^1^H NMR spectra of all investigated plasticizers in non-deuterated n-hexane at a concentration of 1 vol. %. All spectra have been referenced to the signal of the n-hexane (0.8 ppm) peak. Figure S4: ^1^H-NMR (a) *T*_1_ and (b) *T*_2_ relaxation times of TOTM and DIBP at all investigated concentrations measured at 40 MHz. For each concentration, the reported relaxation times are the average of three measurements. Figure S5: ^13^C spectra of DIBP at various concentrations recorded at 40 MHz. The asterisks correspond to the signals from n-hexane. Figure S6: ^1^H NMR spectra of a 0.05 mg mL^−1^ DIBP solution in non-deuterated n-hexane in a stacked plot. SNRs at 3.5 ppm are 5.6, 8.5, and 8.7 for 512, 1024, and 2048 scans, respectively. SNRs at 7 ppm are 6.1, 7.0, and 7.5 for 512, 1024, and 2048 scans, respectively. Table S1: Results of gravimetric analyses of unknown PVC samples used for solvent extraction discussed in Section 2.3. Table S2: Mean results from each sample displayed in Table S1 and individual results from CDCl_3_ and n-hexane extraction.
