# Supplementary material for: Compact NMR Spectroscopy for Low-Cost Identification and Quantification of PVC Plasticizers
Source: Molecules. 2021 Feb 25;26(5):1221. doi: 10.3390/molecules26051221 (PMC7956471; doi:10.3390/molecules26051221)
Supplement: Supplementary file 1 [file molecules-26-01221-s001.pdf]

Supporting Information

Compact NMR Spectroscopy for Low-Cost Identification and Quantification of PVC Plasticizers

Anton Duchowny[1] and Alina Adams*[1]

[1] Institut für Technische und Makromolekulare Chemie, RWTH Aachen University, Templergraben 55, 52056 Aachen, Germany
* Correspondence: Alina.Adams@itmc.rwth-aachen.de; Tel.: (+49)-241-8026428

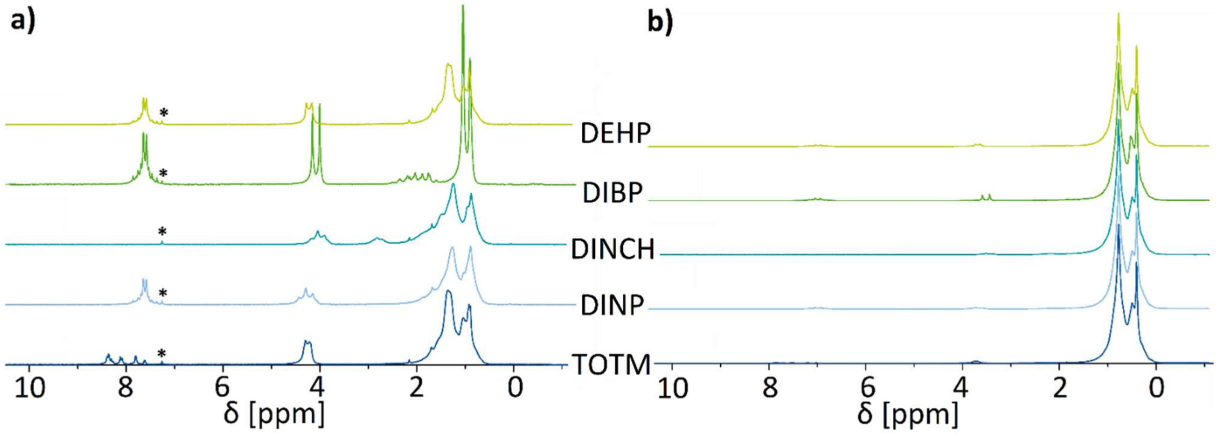

Fig. S1. 40 MHz ^{1}H NMR spectra of the investigated plasticizers at concentrations of 10 vol.% in a) deuterated chloroform and b) non-deuterated n-hexane without zoom. Spectra have been referenced to the residual deuterated chloroform (signal marked with asterisk at 7.26 ppm) and n-hexane (0.8 ppm) peaks, respectively.

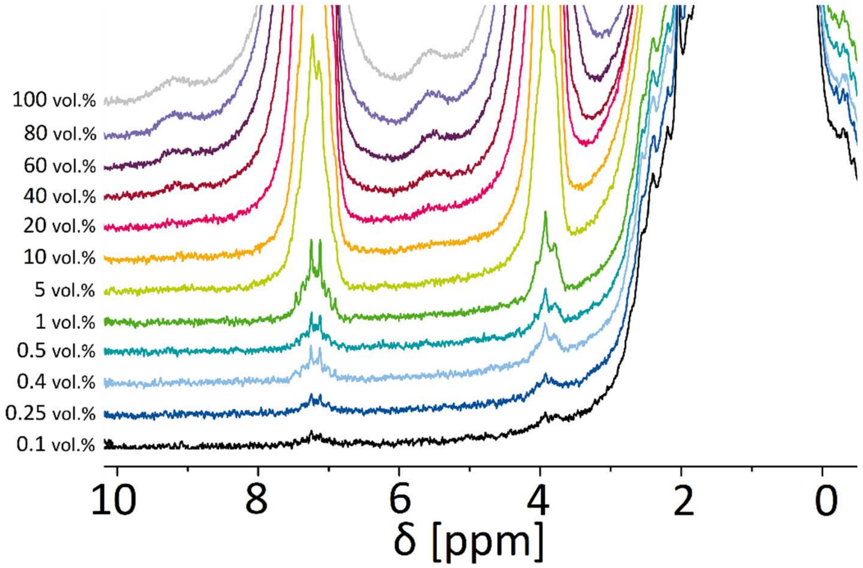

Fig S2. 40 MHz ^{1}H NMR spectra of DINP in non-deuterated hexane at varying concentrations. All spectra have been referenced to the signal of the n-hexane (0.8 ppm) peak. The specific resonances at around 7 ppm can be observed even at concentrations as low as 0.1 vol.% with only 4 scans.

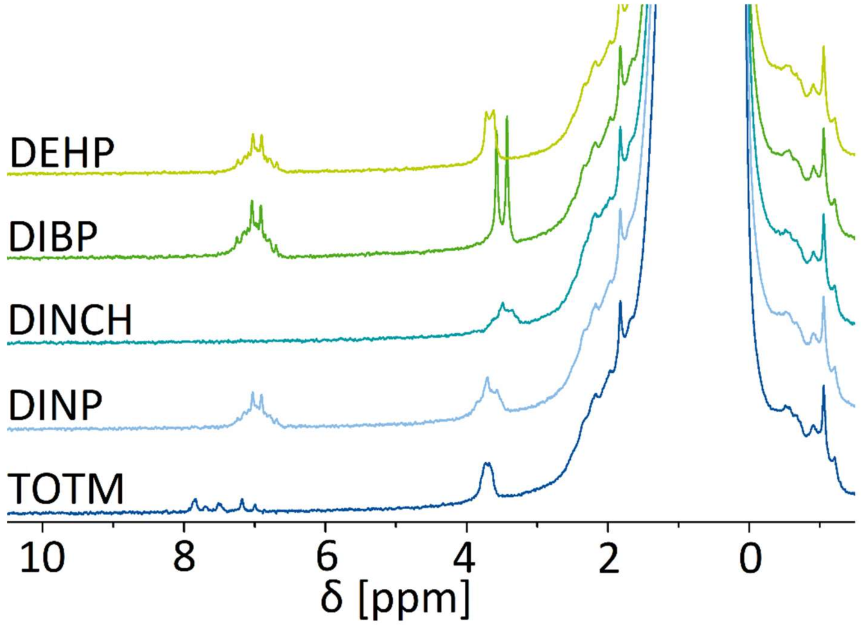

Fig S3. 40 MHz ^{1}H NMR spectra of all investigated plasticizers in non-deuterated n-hexane at a concentration of 1 vol. %. All spectra have been referenced to the signal of the n-hexane (0.8 ppm) peak.

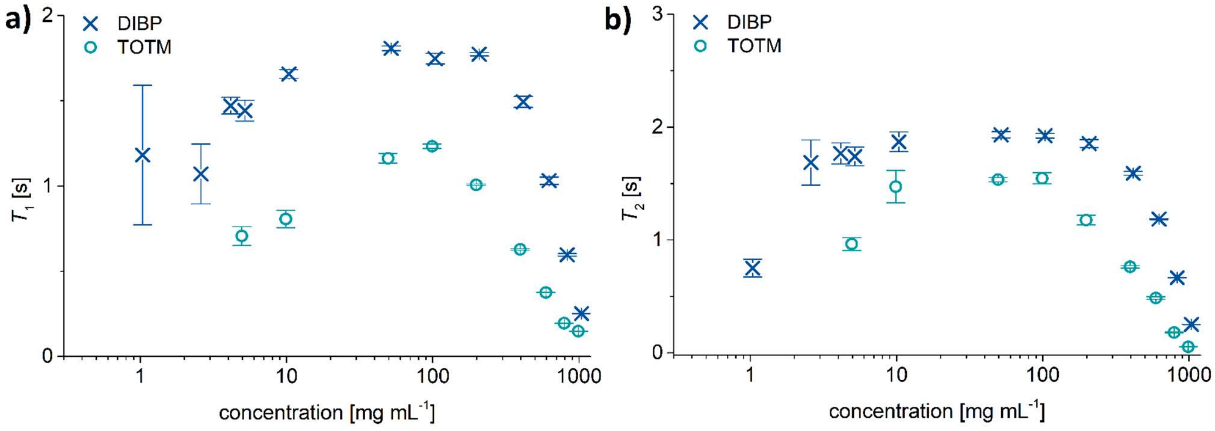

Fig S4. ^{1}H-NMR a) T_1 and b) T_2 relaxation times of TOTM and DIBP at all investigated concentrations measured at 40 MHz. For each concentration, the reported relaxation times are the average of three measurements.

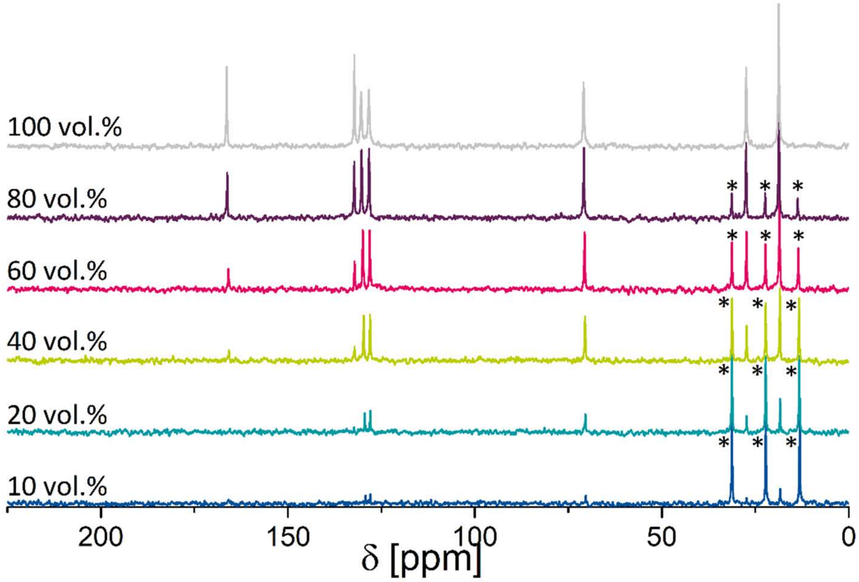

Fig S5. ^{13}C spectra of DIBP at various concentrations recorded at 40 MHz. The asterisks correspond to the signals from n-hexane.

Table S1. Results of gravimetric analyses of unknown PVC samples used for solvent extraction discussed in section 2.3.

sample	initial sample mass [g]	sample mass after extraction and drying [g]	mass difference [g]	mass difference [wt.%]
1a CDCl3	130.7	82.9	47.8	36.57%
1a C6	130.1	82.6	47.5	36.51%
1b CDCl3	131.2	81.9	49.3	37.58%
1b C6	131.7	82.4	49.3	37.43%
1c CDCl3	130.2	82.4	47.8	36.71%
1c C6	120.2	74.1	46.1	38.35%
2a CDCl3	144.5	107.4	37.1	25.67%
2a C6	143.9	107.7	36.2	25.16%
2b CDCl3	144.3	105.6	38.7	26.82%
2b C6	142.5	107	35.5	24.91%
2c CDCl3	137.1	102.7	34.4	25.09%
2c C6	116.7	88.2	28.5	24.42%
3a CDCl3	198.6	187.3	11.3	5.69%
3a C6	192.4	177.6	14.8	7.69%
3b CDCl3	194.9	181.5	13.4	6.88%
3b C6	195.3	180.6	14.7	7.53%
3c CDCl3	163.4	156.3	7.1	4.35%
3c C6	183.5	170.7	12.8	6.98%
4a CDCl3	154.5	102.5	52	33.66%
4a C6	154.7	104.5	50.2	32.45%
4b CDCl3	150.8	99.9	50.9	33.75%
4b C6	152.6	103.4	49.2	32.24%
4c CDCl3	146.9	98.7	48.2	32.81%
4c C6	145.4	98.5	46.9	32.26%
5a CDCl3	304.9	265.7	39.2	12.86%
5a C6	305.3	266.9	38.4	12.58%
5b CDCl3	303.8	268.4	35.4	11.65%
5b C6	301.7	264.7	37	12.26%
5c CDCl3	300.4	265.8	34.6	11.52%
5c C6	283.3	250.2	33.1	11.68%

Table S2. Mean results from each sample displayed in Table S1 and individual results from $CDCl_3$ and n-hexane extraction.

sample	mean wt%	standard deviation	standard deviation [%]	mean wt% of CDCl3 extraction	standard deviation of CDCl3 extraction	standard deviation of CDCl3 extraction [%]	mean wt% of n-hexane extraction	standard deviation of n-hexane extraction	standard deviation of n-hexane extraction [%]
1	33.5%	0.063	18.7%	33.3%	0.066	19.9%	33.6%	0.074	21.9%
2	19.1%	0.097	50.6%	19.2%	0.117	61.1%	19.0%	0.098	51.6%
3	15.3%	0.138	90.1%	15.0%	0.162	108.6%	15.7%	0.146	93.0%
4	26.1%	0.104	39.7%	26.5%	0.118	44.6%	25.7%	0.114	44.2%
5	11.8%	0.003	2.8%	11.6%	0.001	0.8%	12.0%	0.004	3.4%

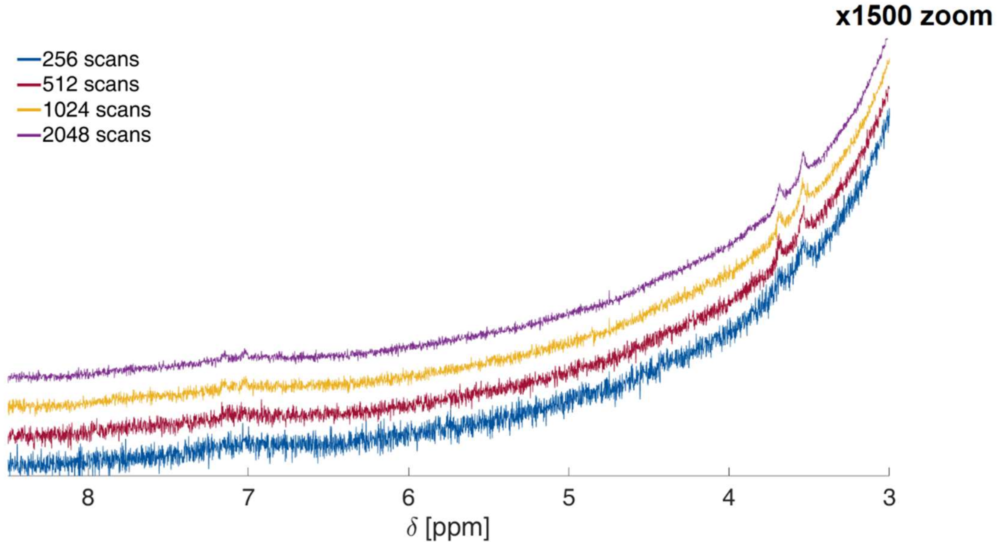

Fig S6. ^{1}H NMR spectra of a 0.05 mg mL^{-1} DIBP solution in non-deuterated n-hexane in a stacked plot. SNRs at 3.5 ppm are 5.6, 8.5, and 8.7 for 512, 1024, and 2048 scans, respectively. SNRs at 7 ppm are 6.1, 7.0, and 7.5 for 512, 1024, and 2048 scans, respectively.
